# Supplementary material for: Front-Line Tyrosine Kinase Inhibitors in Pediatric Chronic Myeloid Leukemia: A Study on Efficacy and Safety
Source: Cancers (Basel). 2023 Jul 29;15(15):3862. doi: 10.3390/cancers15153862 (PMC10416896; doi:10.3390/cancers15153862)
Supplement: Supplementary file 1 [file cancers-15-03862-s001.zip › cancers-2515973-supplementary.pdf]

Supplementary Table S1. Comparison of anthropometric profiles of chronic myeloid leukemia patients treated with imatinib and dasatinib

	IMAT (*n*=33)	DSAT (*n*=18)	*P*
Age, *years*			
TKI start	12.1±4.09	11±5.13	*0.428*
FU 1	13.4±4.12	11.9±5.18	*0.285*
FU 2	14.3±4.1	13.4±4.81	*0.535*
Height SDS			
TKI start	0.14±1.32	-0.27±0.81	*0.381*
FU 1	-0.27±1.23	0.05±1.02	*0.297*
FU 2	-0.44±1.14	-0.48±1.14	*0.979*
Δ Height SDS			
TKI start-FU 1	-0.41±0.63	-0.22±0.37	*0.282*
FU 1-FU 2	-0.07±0.23	-0.27±0.24	*0.035*
TKI start-FU 2	-0.49±0.79	-0.51±0.56	*0.95*
Weight SDS			
TKI start	-0.11±1.41	-0.36±0.73	*0.877*
FU 1	-0.83±2.24	-0.04±0.63	*0.182*
FU 2	0.03±1.33	-0.26±0.89	*0.437*
Δ Weight SDS			
TKI start-FU 1	-0.72±2.68	0.31±0.43	*0.138*
FU 1-FU 2	0.92±2.84	-0.17±0.32	*0.22*
TKI start-FU 2	0.21±0.98	0.15±0.33	*0.81*
BMI SDS			
TKI start	-0.23±1.31	-0.68±0.71	*0.218*
FU 1	-0.57±3.58	-0.11±0.59	*0.612*
FU 2	0.28±1.27	-0.04±0.71	*0.406*
Δ BMI SDS			
TKI start-FU 1	-0.34±3.95	0.57±0.64	*0.373*
FU 1-FU 2	0.86±4.14	-0.07±0.38	*0.471*
TKI start-FU 2	0.55±0.96	0.53±0.34	*0.936*

All values are expressed as mean ± standard deviation.

BMI, body mass index; DSAT, dasatinib group; FU, follow up; IMAT, imatinib group; SDS, standard deviation score; TKI, tyrosine kinase inhibitor
